# Biomechanical Comparative Evaluation of Atrophic Edentulous Maxilla Rehabilitation Using the All-on-Six Concept with Zygomatic Implant and Tilted Implant as Posterior Implant—Finite Element Analysis

**DOI:** 10.3390/dj13120600

**Published:** 2025-12-15

**Authors:** Abhay Datarkar, Neha G. Pawar, Prashant K. Pandilwar, Varsha S. Manekar, Prasad Godase, Eitan Mijiritsky

**Affiliations:** 1 Department of Oral and Maxillofacial Surgery, Govt Dental College & Hospital, Nagpur 440003, Maharashtra, India; abhaydatarkar@yahoo.com (A.D.); drneha7758@gmail.com (N.G.P.); prashant.pandilwar@rediffmail.com (P.K.P.); varshamanekar67@gmail.com (V.S.M.); prasadgodase@gmail.com (P.G.); 2 Department of Head and Neck Surgery and Maxillofacial Surgery, Tel-Aviv Sourasky Medical Center, Tel Aviv University, Tel Aviv 6971915, Israel

**Keywords:** all-on-six implant, atrophic maxilla, biomechanical evaluation, deformation, finite element analysis, full mouth rehabilitation, tilted implants, von Mises stress, zygomatic implant

## Abstract

**Background:** All-on-six implants are a reliable and efficient full-arch restoration option in patients with atrophic maxilla. **Aim:** The present study aimed to evaluate and compare the distribution of stress surrounding implants and adjacent osseous structures by utilizing all-on-six tilted and zygomatic methodologies in the maxillary region by using finite element (FEA) analysis. **Materials and Methods:** Two finite element models were constructed with CT images of 50-year-old female. Model 1 was constructed with zygomatic implants, while model 2 was constructed with tilted implants. A vertical force of 150 N on the anterior component and 300 N on the posterior component were applied and maximum stress and deformation was assessed. **Results:** In the present study, the von Mises stress on the bone and implant overall was higher in the tilted implant model than the zygomatic implant model. **Conclusions:** The findings of the present study suggests that zygomatic implants help distribute occlusal loads more favourably, especially under horizontal and combined loading conditions in an all-on-six configuration.

## 1. Introduction

Various methods of bone augmentation have been described for reconstructing atrophic maxilla. Among them are autogenous block bone grafting; onlay and interpositional bone grafts (smile osteotomy); guided bone regeneration (GBR) with a particular graft; ridge splitting or expansion technique; osteotomies of the ridge or the jaws; distraction osteogenesis; microvascular free flaps; and Le Fort 1 osteotomies combined with bone grafting. Each of these methods has its advantages and disadvantages [1].

Rehabilitation of the atrophic maxilla, particularly in Bedrossian Classification Zone 1 [2], presents a significant clinical challenge for implant-supported prostheses. Traditionally, the all-on-four concept [3] has been widely used to manage such cases. However, approaches suggest the use of an all-on-six concept, involving either four axial and two tilted implants, or a configuration consisting of four axial implants and two zygomatic implants [4,5].

In prosthetic rehabilitation of completely edentulous patients, the “all-on-six” implant technique is a viable option providing functional and esthetic rehabilitation with fewer implants. The strategic placement of six implants often eliminates the need for bone grafting, making it suitable for patients with moderate bone loss who might not qualify for traditional implants [6,7].

Moreover, in the “all-on-six” technique, implants are intentionally angled primarily to obtain anchorage from denser cortical bone areas, increasing primary stability and avoiding critical areas like the maxillary sinus or inferior alveolar nerve [8]. Additionally, tilted implants allow a wider anterior–posterior spread of the implant support, leading to better biomechanical load distribution and reduced stress on the bone and prosthesis [9]. On the other hand, zygomatic implants are especially suitable in severely atrophic maxillae, where bone volume is insufficient for conventional implants. They obtain anchors from the dense zygomatic bone, bypassing the need for sinus augmentation or bone grafting, thus reducing surgical morbidity and treatment time [8,9].

The literature reveals that angulation of the implant, loading direction, different internal connections, and type of bone influences the von Mises stress in the respective peri implant area [10,11]. Higher stress around tilted implants may increase the risk of bone resorption [12].

Considering the high utilization of the all-on-six implants in prosthetic rehabilitation of completely edentulous patients, it is necessary to evaluate the biomechanical response of zygomatic and tilted implants under masticatory loads to anticipate the outcomes and changes in the maxillofacial complex caused by the implants and masticatory forces. Understanding the stress distribution and displacement patterns of the all-on-six implants and maxillofacial complex under masticatory loads can aid in improved selection and planning with respect to implant material, length, diameter, angulation, and other factors.

The present study aimed to evaluate biomechanically the distribution of stress surrounding implants and adjacent osseous structures by utilizing the all-on-six concept with zygomatic and tilted implants as posterior implants in rehabilitation of atrophic edentulous maxilla.

## 2. Materials and Methods

**Study design:** Finite Element Analysis

**Study Setup:** Conducted in the Department of Oral And Maxillofacial Surgery of our institution.

**Ethical standards**: Informed consent was obtained prior to the start of the study. The study was conducted in accordance with the principles outlined in the Declaration of Helsinki. Ethical committee name: Institutional Ethical Committee, Government Dental College and Hospital, Nagpur. Approval Code: IEC/GDCHN/35/2025. Approval Date: 27 January 2025.

**Study Model**

- Model 1: Zygomatic implants as posterior implants and four axial implants in anterior maxilla
- Model 2: Tilted implants as posterior implants and four axial implants in the anterior maxilla.

**Steps of method**

### 2.1. Case Selection

A three-dimensional finite element model of the maxilla was developed using a CT scan of a 50-year-old female patient. A high-resolution multi-slice CT scan was performed on the head and neck region using a CT scanner. The voxel size was non-isotropic, with a slice thickness of 1 mm.

### 2.2. DICOM and STL File Conversion

A CT DICOM file was sent to the biomechanical engineer. All data in Digital Imaging and Communications in Medicine (DICOM) format were imported. The CT image was converted into STL file format using BlueSky Bio Software Version 4.12. A 3-dimensional reconstruction of the images was produced with a surface triangulation technique. For this purpose, a redefinition of edges and surfaces from scanned images was conducted in order to build a simplified model covering most of the volume of original geometry.

Throughout the process special care was taken to keep the surfaces of the implant in contact with the bone as faithful as possible to the original. Designing of the implant was performed by a design tool present in the Geometric Freeform 2025 3-D software.

### 2.3. Construction of the Basic Finite Element Model of Maxilla

After constructing the 3D model, it was converted to a finite element model (FEM) using “Freeform’’ 2025 software. Then the Maxilla model was imported in “BlueSky Bio” Software.

In BlueSky Bio, the maxilla model was analyzed and checked. The geometry data were imported into a generally accepted and already commercially available, FEM programme, ANSYS WORKBENCH 2022R1.

The properties of the bone were assumed to be isotropic, homogenous, and linearly elastic. The behaviour of the bone was characterized by two constants (Young’s modulus and Poisson ratio) (Table 1).

### 2.4. Construction of Maxillary Model with Implants

The dimensions and specifications of the different implants were entered into the software and three-dimensional models of the implants along with the maxilla were created with exact geometry. Zygomatic and conventional implants were inserted into the craniofacial model.

FEA models of zygomatic and conventional tilted implants were constructed 3-dimensionally based on the dimensions from the manufacturer’s catalogue.

The length of the implants was selected according to anatomical contours:

Zygomatic implant: 4.2 × 45 mm.

Tilted posterior implant = 4.2 × 16 mm.

In both models, the dimension of the anterior implant was 3.5 × 10 mm in the central incisor region and 3.75 × 11.5 mm in the canine region. These dimensions were chosen because of the reduced alveolar bone width in the anterior region.

The implants were placed in the skull model, resulting in maximum contact with the bone. The apices of the posterior zygomatic implants were placed as close to the external surface of the zygomatic bone as possible or just extruding through the surface.

All models were imported as different files and 2 model groups were created as model 1 (zygomatic implants as posterior implants and four axial implants in the anterior maxilla) and model 2 (tilted implants as posterior implants and four axial implant in the anterior maxilla).

### 2.5. Occlusal Loading

With an adequate and simplified geometric finite element model and the appropriate material properties, along with the occlusal loading and support conditions, the finite element solver module of the ANSYS software (ANSYS WORKBENCH 2022R1) carried out the mathematical procedure, and displacement and stress values for each node as well as element were obtained.

A vertical force of 150 N on the anterior component and 300 N on the posterior component were applied, simulating load transfer from the prosthesis. It was considered that the force was uniformly distributed through the connector to the component of implants. The response was recorded as per the software settings for the maximum stress on the underlying remaining bone and on respective implants along with displacement and deformation.

### 2.6. Finite Element Analysis

On application of the occlusal forces, the total structural deformation of the implants, along with the bone and von Mises stresses generated, were obtained and recorded for each configuration of implants using the computerized software. By analyzing the VM stresses predicted by the model for each configuration and total structural deformation of the implant in each configuration, we could analyze which implant configuration provides the greatest stability.

VM stress is a value used to determine whether a given material will yield or fracture under a given load. If the VM stress is greater, the material is expected to yield or fracture. Hence, configuration with the lowest relative VM stress and the least structural deformation were likely to be the most stable.

## 3. Results

**Figure 1:**
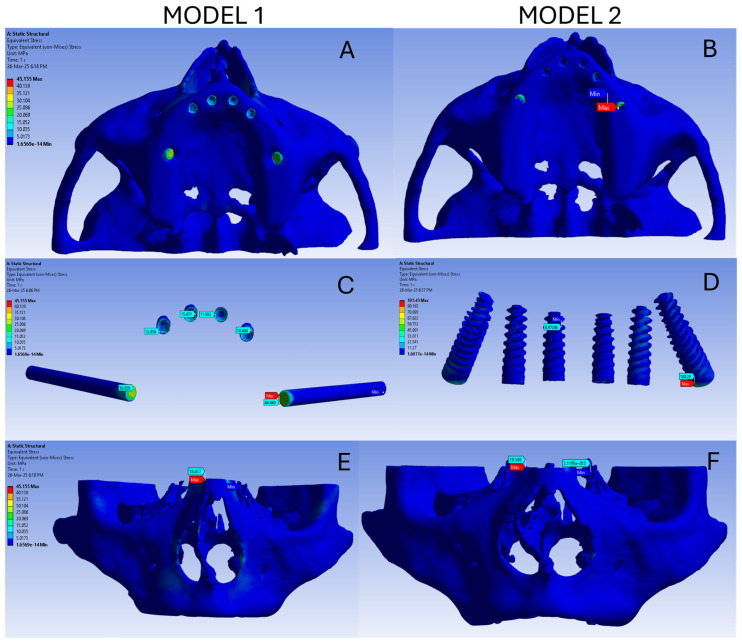
(**A**) Overall stress in model 1; (**B**) Overall stress in model 2; (**C**) Stress on the implant of model 1; (**D**) Stress on the implant of model 2; (**E**) Stress on the bone of model 1; (**F**) Stress on the bone of model 2.

The stress and deformation in both the models are summarized in Table 2. The overall stress in model 1 ranges from 1.65 × 10^−14^ MPa to 45.155 MPa, as represented by the blue colour-coded image (Figure 1A). In model 1 the stress on the implant ranged from 8.02 × 10^−2^ MPa to 44.869 MPa and high stress was noted in the neck of the screw. Among the six screws, the highest stress was observed in the zygomatic implants followed by the anterior implant (Figure 1C). The stress on the bone in model 1 ranged from 1.65 × 10^−14^ MPa to 18.418 MPa and is represented in Figure 1E.

**Figure 2:**
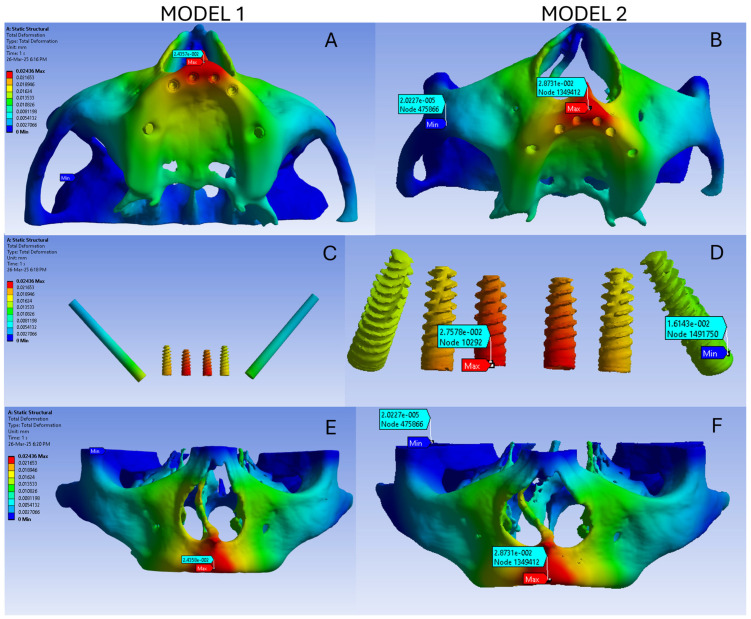
(**A**) Overall deformation in model 1; (**B**) Overall deformation in model 2; (**C**) Deformation of the implants in model 1; (**D**) Deformation of the implants in model 2; (**E**) Deformation of the bone in model 1; (**F**) Deformation of the bone in model 2.

The overall deformation in model 1 ranges from 0 to 0.024 mm, as represented by the colourcoded image (Figure 2A). The highest deformation in model 1 was noted as the red colour region in the anterior maxilla. The deformation reduces gradually posteriorly and is represented as red to yellow rather than to a green colour (Figure 2C). In model 1 the deformation of the implant ranges from 0.0036 mm to 0.023 mm and high deformation is noted in the anterior implant in the incisor region followed by the anterior implant in the canine region, and the least deformation is noted in the zygomatic implants (Figure 2E). The deformation of the bone in model 1 ranges from 0 to 0.024 mm and is represented in Figure 2E. The highest deformation occurred in the premaxillary region.

The overall stress in model 2 ranges from 1.6077 × 10^−14^ MPa to 101.43 MPa, as represented by the blue colour-coded image (Figure 1B). In model 2, the stress on the implant ranges from 0.97 MPa to 100.29 MPa and is coded blue. All six screws show minimal stress in the tilted implant model (Figure 1D). The stress on the bone in model 2 ranges from 1.6077 × 10^−13^ MPa to 19.54 MPa and is represented in Figure 1F.

The overall deformation in model 2 ranges from 0 to 0.024 mm, as represented by the colour-coded image (Figure 2B). The highest deformation is noted as a red colour region in the anterior maxilla. The deformation reduces gradually posteriorly and is represented as red to yellow rather than to a green colour. Deformation is also noted in the zygomatic bone, maxilla, and nasal spine (Figure 2D). In model 2, the deformation of the implant ranges from 0.016 mm to 0.027 mm and high deformation is noted in the anterior implant in the incisor region followed by the anterior implant in the canine region, and the least deformation is noted in tilted implants (Figure 2D). The deformation of the bone ranges in model 2 from 0 to 0.028 mm and is represented in Figure 2F. The highest deformation occurs in the premaxillary region. The von Misses stress on both the models is comparable. Also the total deformation and bone deformation of both the models is comparable.

## 4. Discussion

Finite element analysis (FEA) is a computational technique widely used to evaluate stress distribution patterns. FEA is a numerical simulation technique used to predict how objects or structures behave under various physical conditions such as forces, heat, pressure, or vibration [13,14]. In the present study, FEA analysis was used to assess and compare the stress and deformation pattern in all-on-six dental implant systems with zygomatic and tilted implants under masticatory forces.

In the present study, the von Mises stress on the bone and implant as well as overallstress was higher in the tilted implant model than the zygomatic implant model. These findings were in accordance with study by Tezerişener et al. [15] who observed that higher stress values were observed in both cortical and trabecular bone around the 45°-tilted posterior implants while the lowest stress was determined in the model including anterior dental implants combined with zygomatic implants. However, Tezerişener et al. had investigated the all-on-four concept, which is in contrast to the present study.

In the current study, the highest maximum principal stress in cortical bone was observed in model 2, suggesting that tilting the posterior implant by 45° caused an increase in tensile stress around this implant compared to the first model. Similar findings were observed by Pellizzer et al. [16]. They reported an increase in stress accumulation as the angle of the implant increased.

In the present study, in model 1, high stress was noted in the neck of the screw. This is in accordance with studies by Silva et al. [17] and Tezerişener et al. [15]. Zygomatic implants combined with two to four conventional implants have been considered to be the ideal treatment option if the bone volume in the anterior maxilla is sufficient [18,19]. Another study revealed that an implant system incorporating one zygomatic implant with multiple standard implants showed lower maximum principal stress values in the maxilla rather than with two zygomatic implants or only tilted implants [20].

In the present study, the total deformation values ranged from 0 to 0.024 mm in model 1 and up to 0.028 mm in model 2; implant deformation was up to 0.023 mm in model 1 and 0.027 mm in model 2. Similarly, in Saber et al.’s study [21], the results showed low deformation magnitudes. Additionally, their study also revealed that increasing the inclination in posterior implants resulted in a reduction in cantilever length and maximum stress declined in both cancellous and cortical bone. These findings indicate that both implant designs maintain structural integrity under physiological masticatory loads without excessive displacement detrimental to osseointegration [21].

The findings of the present study suggests that zygomatic implants help distribute occlusal loads more favourably, especially under horizontal and combined loading conditions. These findings are in accordance with a study by Jaiswal et al. [22] who revealed that the all-on-six treatment concept showed the most favourable biomechanical behaviour with consistently better stress measurements on the cortical bone and implants than those obtained from the all-on-four configuration.

In reviewing the literature, no data was found that compared the biomechanical behaviour of zygomatic implants and the tilted implant in an all-on-six system in prosthetic rehabilitation of atrophic maxilla. The results of the present study indicate that the zygomatic implant reduces biomechanical stress on maxillary bones compared to tilted implants and thereby eliminate the unnecessary bone resorption due to excess stress distribution. The increase in anteroposterior span of the prosthesis will give a better masticatory force distribution and overall satisfaction to patients.

## 5. Limitations

The present study has few limitations. Firstly, the FEA models are simplifications of complex biological systems and rely on assumptions such as isotropic, homogeneous, and linearly elastic material properties for bone and implant components, which do not fully replicate the anisotropic and heterogeneous nature of human bone. Secondly, the models typically consider ideal osseointegration with 100% implant-bone contact, which may not correspond to clinical reality where variations in bone quality, healing, and implant stability exist. Thirdly, the loading conditions in FEA are usually static and simplified (axial or oblique forces) and may not reflect the multidirectional and dynamic masticatory forces encountered in vivo. Lastly, software limitations and mesh quality may introduce computational errors affecting result reliability.

## 6. Future Perspective

In future, development of dynamic loading conditions mimicking actual mastication cycles and parafunctional habits need to be integrated into simulations for more comprehensive biomechanical evaluation. Additionally, FEA models constructed with various degrees of tilt, implant length, and diameter should be investigated to draw a conclusion.

## 7. Conclusions

All-on-six full-arch maxillary rehabilitation is a biomechanically favourable and minimally invasive approach for completely edentulous patients with compromised bone anatomy. The present finite element analysis demonstrates that zygomatic implants, when combined with standard dental implants in an all-on-six system, can effectively reduce stress concentrations in the atrophic maxillary bone compared to configurations on tilted implants, offering superior load distribution in full-arch rehabilitations.

## Figures and Tables

**Table 1:** Young’s modulus and Poisson ratio.

Material	Young’s Modulus (MPa)	Poisson’s Ratio	Tensile Yield Strength (MPa)
Titanium	110,000	0.35	834
Cortical bone	13,700	0.3	114
Cancellous bone	1370	0.3	52

**Table 2:** Stress and deformation in model 1 and model 2.

		MODEL 1		MODEL 2	
Sr. No.	Sensor Name	Minimum	Maximum	Minimum	Maximum
1	Von Mises Stress Overall (MPa)	1.65 × 10^−14^ MPa	45.155 MPa	1.6077 × 10^−14^ MPa	101.43 MPa
2	Von Mises Stress Implant (MPa)	9.02 × 10^−2^ MPa	44.869 MPa	0.97 MPa	100.29 MPa
3	Von Mises Stress Bone (MPa)	1.65 × 10^−14^ MPa	18.418 MPa	1.6077 × 10^−13^ MPa	19.54 MPa
4	Total Deformation (mm)	0 mm	0.024 mm	0 mm	0.028 mm
5	Implant Deformation (mm)	0.0036 mm	0.023 mm	0.016 mm	0.027 mm
6	Bone Deformation (mm)	0 mm	0.024 mm	0 mm	0.028 mm

## Data Availability

The original contributions presented in this study are included in the article. Further inquiries can be directed to the corresponding author.
